# Successful treatment of acute promyelocytic leukemia in a 92-year-old man using all-trans retinoic acid combined with oral arsenic: A case report

**DOI:** 10.1097/MD.0000000000026144

**Published:** 2021-06-04

**Authors:** Xiaowei Shi, Shuangyue Li, Shanhao Tang, Ying Lu

**Affiliations:** Department of Hematology, Yinzhou People's Hospital, Ningbo, Zhejiang, P.R. China.

**Keywords:** acute promyelocytic leukemia, all-trans retinoic acid, elderly patients, no chemotherapy, realgar-indigo naturalis formula

## Abstract

**Rationale:**

Acute promyelocytic leukemia is a special subtype of acute myeloid leukemia. The incidence of early death and complications is high. An oral regimen of all-trans retinoic acid combined with the realgar-indigo naturalis formula (RIF) without chemotherapy has provided a new strategy for the treatment of these patients.

**Patient concerns:**

A 92-year-old male patient was admitted to the hospital due to fatigue and oral bleeding. He had no fever or lung infection. Routine blood test showed white blood cell count 1.0 ×109/L, hemoglobin 100 g/L, and platelets 21 × 10^9^/L. Coagulation function indicated fibrinogen 1.02 g/L and D-dimer 2360 ng/mL. And 28% abnormal promyelocytes were observed in peripheral blood.

**Diagnosis:**

A bone marrow morphologic, immunophenotypic, cytogenetic, and molecular examination was performed. Routine bone marrow examination showed active proliferation of nucleated cells, with promyelocytes accounting for 91%; immunophenotyping revealed an early myeloid cell population, accounting for approximately 82.4% of all cells.

**Interventions:**

From February 15, 2020, 25 mg/m^2^ all-trans retinoic acid was orally administered daily. After the fusion gene result was obtained, oral administration of 60 mg/kg RIF daily began since February 18, 2020. The combination of the 2 agents was given until March 16, 2020. Oral administration of 25 mg/m^2^ retinoic acid daily began from March 20, 2020 for 2 weeks, and oral administration of 60 mg/kg RIF daily lasted for 4 weeks as the consolidation therapy. During the treatment, the proportion of promyelocytes in peripheral blood, white blood cell count, platelets, coagulation function, liver function, and QT interval were monitored.

**Outcomes:**

Oral retinoic acid and oral RIF were given without chemotherapy and the patient achieved bone marrow remission after 1 month, and molecular remission was achieved 2 months later. In the early stage of acute promyelocytic leukemia, combined thrombocytopenia and disseminated intravascular coagulation may develop. Platelet and fresh frozen plasma infusion were proactively given until platelets were stabilized above 30 × 10^9^/L, and the coagulation function returned to normal.

**Lessons:**

The regimen was safe and effective, and subsequent treatment did not require hospitalization, which helped to improve the patient's quality of life.

## 1 Introduction

Acute promyelocytic leukemia (APL) is a special subtype of acute myeloid leukemia. It is characterized by a translocation between chromosomes 15 and 17, which leads to the fusion of the promyelocytic leukemia protein gene and the retinoic acid receptor alpha gene. At present, the complete remission rate is 90% to 100% in patients receiving a regimen based on all-trans retinoic acid (ATRA) and arsenic trioxide.^[1,2]^ The only oral arsenic agent, realgar-indigo naturalis formula (RIF), was launched in China in 2009. Based on the clinical experience in recent years, RIF not only has the equal clinical effect as injections, but also has a higher safety profile.^[3]^ Therefore, it has been incorporated into the Chinese Acute Promyelocytic Leukemia Treatment Guidelines since 2014.^[4,5]^ Zhu et al ^[6]^ proposed that an oral regimen of ATRA combined with RIF without chemotherapy has become the first-line treatment for Chinese patients with APL, and this regimen can significantly improve the quality of life of patients and reduce the cost of medical treatment.

The early stage of APL is often accompanied by thrombocytopenia and disseminated intravascular coagulation. Elderly patients are prone to develop complications such as infection and bleeding due to underlying conditions and poor tolerance, resulting in a high mortality rate.^[7,8]^ Although treatment efficacy of APL has been considerably improved, the overall survival rate of elderly patients is still significantly lower than that of young patients,^[9]^ and there are still substantial difficulties in the treatment of elderly patients. Here, we reported a 92-year-old patient who had been referred in a timely manner. He received an oral regimen of ATRA combined with RIF without chemotherapy for 28 days. After bone marrow re-examination, complete remission was achieved.

## 2 Case description

On February 15, 2020, a 92-year-old male patient was admitted to the hospital due to fatigue and oral bleeding. He had no fever or lung infection. Routine blood test showed white blood cell count 1.0  × 10^9^/L, hemoglobin 100 g/L, and platelets 21 × 10^9^/L. Coagulation function indicated fibrinogen 1.02 g/L and D-dimer 2360 ng/mL. And 28% abnormal promyelocytes were observed in peripheral blood. A bone marrow morphologic, immunophenotypic, cytogenetic, and molecular examination was performed on February 26, 2020. Routine bone marrow examination showed active proliferation of nucleated cells, with promyelocytes accounting for 91%; immunophenotyping revealed an early myeloid cell population, accounting for approximately 82.4% of all cells. Partial expression of CD117 and CD34, HLA-DR-, CD13^++^, CD33^++^, partial expression of CD64, CD38^++^, MPO^+^; the copy number of promyelocytic leukaemia (PML)/retinoic acid receptorα (RARα) (L-type) fusion gene was 10,655 copies/10,000 Abl copies; chromosome results were 45, X, −Y [3]/46, XY, t (15; 17) (q22; q21) [1]/46, XY.^[10]^ Overall, the patient was diagnosed as acute promyelocytic leukemia (intermediate risk group).

The patient had a history of hypertension and diabetes, and blood pressure and blood glucose levels were controlled within the normal range by oral antihypertensive drugs and hypoglycemic medications. From February 15, 2020, 25 mg/m^2^ all-trans retinoic acid was orally administered daily. After the fusion gene result was obtained, oral administration of 60 mg/kg RIF daily began since February 18, 2020. The combination of the two agents was given until March 16, 2020. During the treatment, the proportion of promyelocytes in peripheral blood (Table 1), white blood cell count (Table 2), platelets, coagulation function (Table 3), liver function (Table 4), and QT interval (Table 5) were monitored. Platelet and fresh frozen plasma were infused intermittently during the treatment. On February 23, 2020, the white blood cell count was increased significantly. The patient experienced chest tightness, discomfort, and hypothermia. Chest computed tomography showed bilateral lung infections with pleural effusion. Anti-infective treatment was initiated. At the same time, dexamethasone 5 mg q12 h was given and gradually reduced after 3 days to prevent retinoic acid syndromes. White blood cells continued to rise, and low-dose hydroxyurea was given to reduce the number of white blood cells. On March 6, 2020, the number of white blood cells returned to normal, and on March 7, 2020, no promyelocytes were seen in the peripheral blood. Routine bone marrow re-examination on March 18, 2020 showed active proliferation of nucleated cells, with promyelocytes accounting for 2%; the fusion gene PML/RARα (L type) copy number was 92 copies/10,000 abl copies; chromosome results were 45, X, −Y [4]/46, XY.^[11]^

**Table 1:**
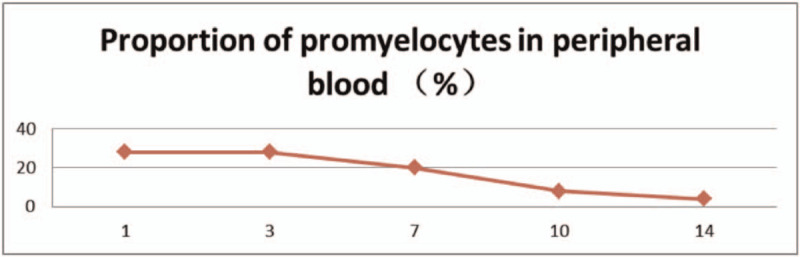
The proportion of promyelocytes in peripheral blood.

**Table 2:**
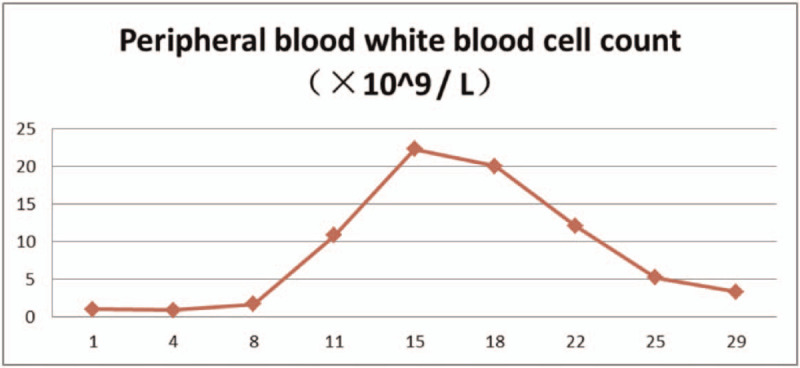
The proportion of white blood cell count in peripheral blood.

**Table 3:**
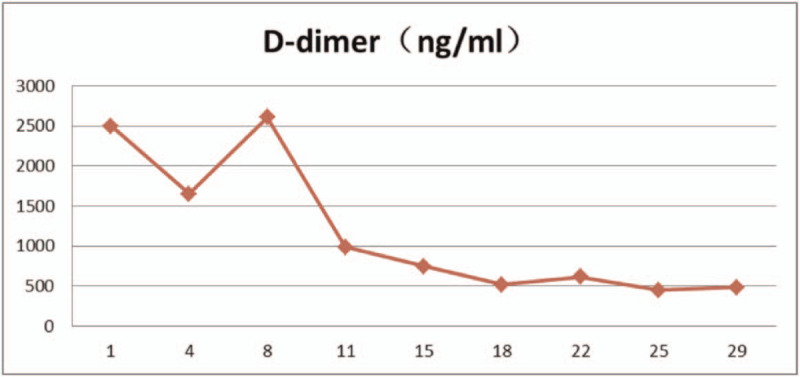
The proportion of white blood cell count in peripheral blood.

**Table 4:**
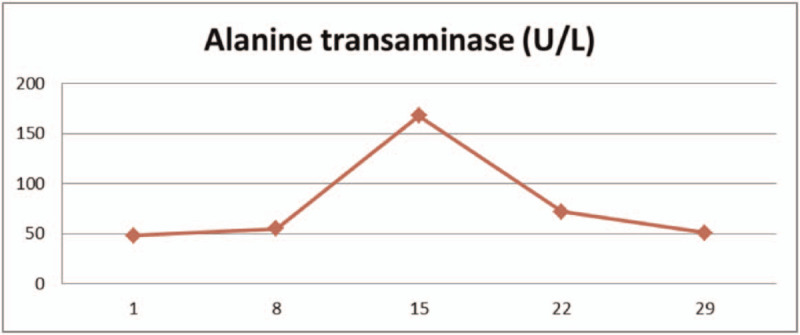
liver function.

**Table 5:**
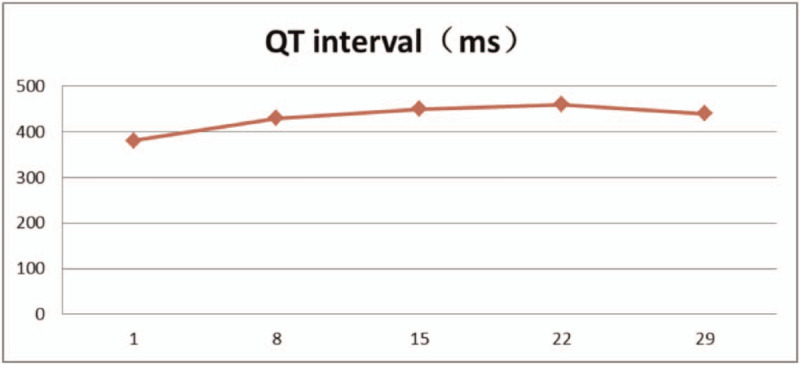
QT interval.

Oral administration of 25 mg/m^2^ retinoic acid daily began from March 20, 2020 for 2 weeks, and oral administration of 60 mg/kg RIF daily lasted for 4 weeks as the consolidation therapy. Routine bone marrow re-examination on April 18, 2020 showed active proliferation of nucleated cells, no promyelocytes; no PML/RARα fusion gene detected; chromosome results were 45, X, −Y [4]/46, XY,^[11]^ XY. The latest follow-up results of the patient was January 17, 2021. The results showed that the patient had normal blood routine, normal human myelogram, negative PML-RARα gene, and normal karyotype (Table 6)

**Table 6:** The follow-up results of the patient.

	Bone marrow promyelocyte percentage (%)	PML/RARα	
Copy number	Chromosome		
February 15, 2020	91	10,655 copies/10,000 abl copies	45, X, −Y[3]/46, XY, t(15;17)(q22;q21)[1]/46, XY[12]
March 18, 2020	2	92 copies/10,000 abl copies	45, X, −Y[4]/46, XY[11]
April 18, 2020	0	0	45, X, −Y[4]/46, XY[11]
January 17, 2021.	0	0	46, XY[20]

## 3 Discussion

An elderly patient with new-onset APL was treated successfully. The treatment process had the following characteristics: oral retinoic acid and oral RIF were given without chemotherapy, and the patient achieved bone marrow remission after 1 month, and molecular remission was achieved 2 months later. After the differentiation of peripheral blood leucocytes, dexamethasone was used to prevent retinoic acid syndromes. At the same time, low-dose hydroxyurea was given to reduce the number of leucocytes, so they could successfully pass through the cell differentiation phase. In the early stage of APL, combined thrombocytopenia and disseminated intravascular coagulation may develop. Platelet and fresh frozen plasma infusion were proactively given until platelets were stabilized above 30 × 10^9^/L, and the coagulation function returned to normal.

Acute promyelocytic leukemia is a special subtype of leukemia with a very high cure rate. Induction therapy involves all-trans retinoic acid combined with arsenic trioxide or anthracycline drugs, and the complete remission rate can reach 90% or above. Therefore, even for elderly patients, treatment should not be rejected. However, elderly patients are often complicated with severe bleeding, infections, and multiple organ failures due to underlying conditions, decreased organ function, weakened immune function, and poor tolerance to chemotherapy,^[8]^ resulting in lower overall survival (OS) compared with younger patients.^[12]^ Once the elderly patients are diagnosed with APL, their expectation for survival is generally low, and healing is usually not considered as a treatment goal, nor can they be referred in the first instance to a specialist hospital for initiation of all-trans retinoic acid therapy.^[11]^ This is another factor contributing to the poor overall therapeutic outcomes. A study by Kayser et al ^[10]^ showed that in APL patients aged 70 years or over, the early mortality rate in those treated with all-trans retinoic acid combined with arsenic trioxide was 7%, with a 5-year OS of 75%, whereas the early mortality rate in those treated with retinoic acid combined with chemotherapy was 24%, with a 5-year OS of only 58%. These results indicated that treatment using retinoic acid combined with arsenic trioxide was significantly more effective than the combination of retinoic acid and chemotherapy in terms of early mortality and long-term survival rate in elderly patients. Burnett et al ^[13]^ reached similar conclusions in their study. The 5-year OS and event free survival in elderly patients treated with ATRA combined with arsenic trioxide were better than ATRA combined with chemotherapy. At the same time, treatment using ATRA combined with arsenic trioxide was associated with lower haematological toxicity and a lower risk of secondary infections.^[14]^ The APL0406 study by Cicconi et al ^[15]^ also confirmed these findings. However, liver toxicity and Q-T interval prolongation are more common, especially in elderly patients, and particular attention should be paid to the risks to the heart. Liver function, electrolytes, and electrocardiogram are required to be monitored regularly, to ensure that blood potassium is above 4.0 mmoL/L and that blood magnesium is above 1.8 mg/dL to reduce the risk of Q-T interval prolongation.^[16]^ When the QT interval extends beyond 500 ms, arsenic trioxide should be stopped. After returning to normal, it can be increased by 50% increments to the conventional dose.^[17]^ Recently, the expert group of the International Society of Geriatric Oncology recommended that elderly patients aged 75 or over with low- and middle -risk of APL should receive ATRA combined with arsenic trioxide, and they demonstrated that there is no definitive evidence that dose adjustment is required. The use of anthracyclines should be avoided as much as possible to reduce the side effects of chemotherapy.^[18]^ At present, in China, more than 5000 APL patients have received the treatment of ATRA combined with arsenic trioxide as oral preparation of RIF tablets. The results showed that RIF had the same clinical efficacy as intravenous preparations while the risk of toxic side effects is relatively lower.^[3,19,20]^ When oral arsenic trioxide was compared with intravenous preparations, a recent APL clinical phase III study by Zhu et al^[3]^ found that the incidence of grade 3 to 4 liver dysfunction was 9% versus 14%, and the infection rate was 23% versus 42%. This is the basis for our final choice of ATRA combined with RIF in the treatment of this patient. The early stage of APL is often accompanied by fatal disseminated intravascular coagulation,^[21,22]^ so it is necessary to promote supportive treatment while treating the primary disease, including the infusion of fibrinogen or fresh frozen plasma and platelets. The platelets are maintained above 30 × 10^9^/L to reduce the risk of bleeding.^[23]^ During the induction therapy, the risk of retinoic syndromes in elderly patients with APL is higher than that in young patients,^[24]^ and the risk of retinoic acid syndromes with retinoic acid combined with arsenic trioxide treatment is higher than that with retinoic acid combined with chemotherapy,^[18]^ which is related to the increase of white blood cells during the treatment. Therefore, Stahl and Tallman^[25]^ proposed that all patients treated with this regimen should be given steroid prophylaxis. At the same time, they suggested that oral hydroxyurea 500 mg once a day should be given when the white blood cell count is 10 to 50 × 10^9^/L, and that hydroxyurea 1000 mg once a day should be given when the white blood cell count is over 50 × 10^9^/L, and that the oral administration should be terminated when the white blood cell count is less than 10 × 10^9^/L.

Based on the facts above, we made the correct choice in treatment plan. The patient did not develop severe secondary myelosuppression, septicaemia, haemorrhage, obvious liver or kidney dysfunction during the treatment period, and successful bone marrow remission was achieved. This is the first case report of successful treatment of APL in a patient over the age of 90 using oral retinoic acid and RIF without chemotherapy.

## 4 Conclusion

Overall, the regimen was safe and effective, and subsequent treatment did not require hospitalization, which helped to improve the patient's quality of life. From the treatment of this patient, we highlighted that the opportunity for treatment should not be rejected even in very elderly APL patients.

## Acknowledgments

The authors acknowledge the reviewers for their helpful comments on this paper.

## Author contributions

Shanhao Tang, Ying Lu finished the data analysis, Xiaowei Shi, Shuangyue Li finished the manuscript writing and editing. All authors read and approved the final manuscript.

**Conceptualization:** Ying Lu.

**Data curation:** Xiaowei Shi, Shuangyue Li, Shanhao Tang.

**Writing – original draft:** Xiaowei Shi.
